# Diet-dependent, microbiota-independent regulation of IL-10-producing lamina propria macrophages in the small intestine

**DOI:** 10.1038/srep27634

**Published:** 2016-06-15

**Authors:** Takanori Ochi, Yongjia Feng, Sho Kitamoto, Hiroko Nagao-Kitamoto, Peter Kuffa, Koji Atarashi, Kenya Honda, Daniel H. Teitelbaum, Nobuhiko Kamada

**Affiliations:** 1 Division of Gastroenterology, Department of Internal Medicine, University of Michigan, Ann Arbor, MI, USA; 2 Section of Pediatric Surgery, Department of Surgery, University of Michigan, Ann Arbor, MI, USA; 3 Department of Microbiology and Immunology, Keio University School of Medicine, Tokyo, Japan

## Abstract

Intestinal resident macrophages (Mϕs) regulate gastrointestinal homeostasis via production of an anti-inflammatory cytokine interleukin (IL)-10. Although a constant replenishment by circulating monocytes is required to maintain the pool of resident Mϕs in the colonic mucosa, the homeostatic regulation of Mϕ in the small intestine (SI) remains unclear. Here, we demonstrate that direct stimulation by dietary amino acids regulates the homeostasis of intestinal Mϕs in the SI. Mice that received total parenteral nutrition (TPN), which deprives the animals of enteral nutrients, displayed a significant decrease of IL-10-producing Mϕs in the SI, whereas the IL-10-producing CD4^+^ T cells remained intact. Likewise, enteral nutrient deprivation selectively affected the monocyte-derived F4/80^+^ Mϕ population, but not non-monocytic precursor-derived CD103^+^ dendritic cells. Notably, in contrast to colonic Mϕs, the replenishment of SI Mϕs and their IL-10 production were not regulated by the gut microbiota. Rather, SI Mϕs were directly regulated by dietary amino acids. Collectively, our study highlights the diet-dependent, microbiota-independent regulation of IL-10-producing resident Mϕs in the SI.

Intestinal resident macrophages (Mϕs) are innate immune cells that play a central role in the maintenance of homeostasis in the gastrointestinal (GI) tract^1^,^2^. In addition to their phagocytic and bactericidal roles, intestinal Mϕ produce robust amounts of interleukin (IL)-10, a major immune-regulatory cytokine that maintains mucosal homeostasis^3^,^4^,^5^, and limit excessive immune responses against dietary and bacterial antigens present in the intestinal lumen^4^. Likewise, intestinal Mϕs promote the differentiation and maintenance of regulatory T (T_reg_) cells in the intestine via production of IL-10^2^,^6^,^7^. Thus, intestinal Mϕs both directly and indirectly contribute to the dampening of inflammation in the intestine^1^,^2^,^6^,^7^.

Recent extensive studies have shed more light on the homeostatic regulation of intestinal resident Mϕs. It has become evident that intestinal resident Mϕs are derived from blood Ly6C^hi^CCR2^+^ monocytes in the adult intestine^8^,^9^. Notably, intestinal Mϕs in the adult intestine have a diminished ability to self-renew; rather, intestinal Mϕs are constantly replenished by circulating Ly6C^hi^CCR2^+^ monocytes in a CCL2-CCR2-dependent manner^10^. Although the replenishment of resident Mϕ populations in the colonic mucosa is known to be regulated by the resident microbiota^9^,^10^, little is known about the regulation of resident Mϕs in the small intestine (SI). Since the density of bacteria in the lumen is markedly lower in the SI compared to the colon, it is possible that other factors contribute to the replenishment of resident Mϕs in the SI.

Total parenteral nutrition (TPN) is an essential form of therapy for patients whose GI tract cannot fully absorb nutrients, such as short bowel syndrome^11^,^12^. TPN supplies all daily nutritional requirements, including carbohydrates, fats, vitamins, and micronutrients. TPN is typically administered through an intravenous infusion. Although the therapy is life-sustaining for many, TPN also leads to serious adverse events including mucosal atrophy, loss of epithelial barrier function and frequent systemic infections^13^. We previously reported that human TPN recipients display the same adverse phenotypes as can be seen in a mouse model of TPN^14^,^15^,^16^. Notably, it has been shown that TPN treated mice exhibited impaired production of IL-10 in the SI lamina propria (LP)^16^,^17^. Since the administration of exogenous IL-10 corrects the impairment of epithelial barrier function and restores SI homeostasis^17^,^18^, blunted mucosal IL-10 production is a key driving factor of the adverse phenotypes seen in TPN recipients. However, at present the precise mechanisms by which the lack of enteral nutrients due to TPN causes diminished mucosal IL-10 production remain unclear.

Here, we show that the deprivation of enteral nutrients associated with TPN leads to a decrease in the number of IL-10-producing F4/80^+^CD11b^+^ Mϕs in the SI LP. In contrast to colonic resident Mϕs, the replenishment of SI Mϕ is not regulated by the gut microbiota. Rather, it is regulated by dietary amino acids. Collectively, our study highlights the role of dietary stimulation through the enteral route in the regulation of resident Mϕ homeostasis in the SI. Enteral nutrient deprivation due to TPN leads to a disruption of SI homeostasis through the impairment of IL-10 production by resident Mϕs.

## Results

### TPN results in a marked decline in IL-10-producing F4/80^+^CD11b^+^ macrophages

**Figure 1:**
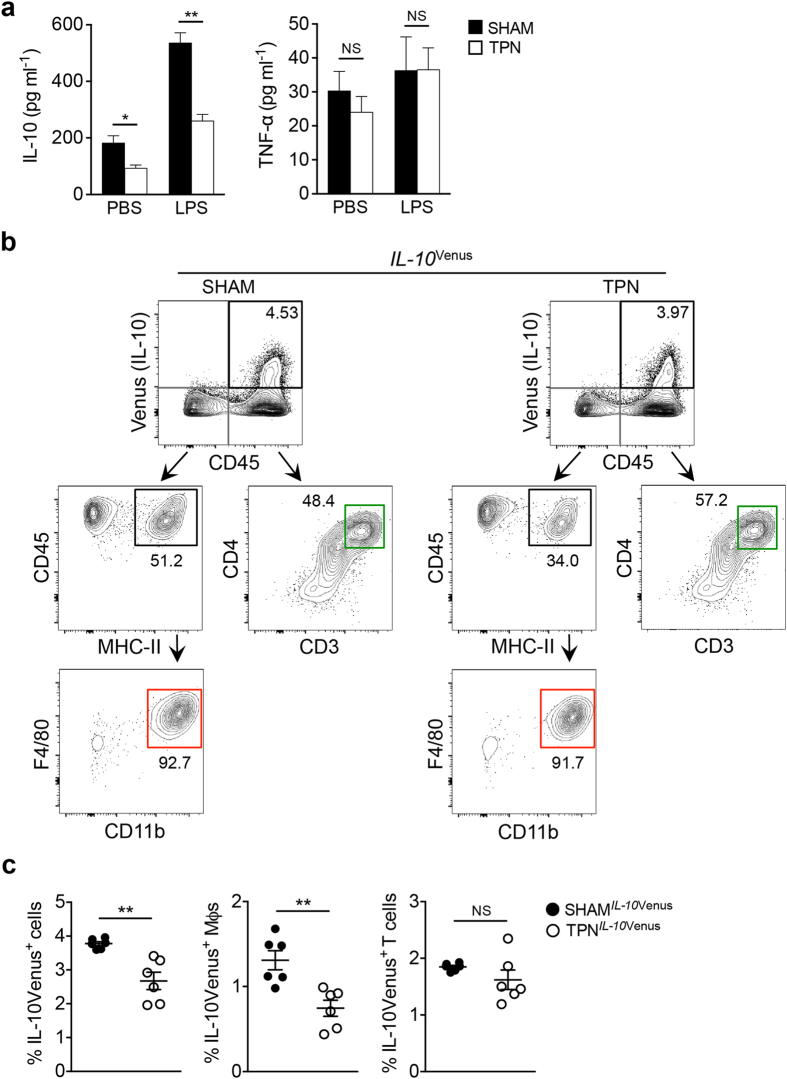
Deprivation of enteral nutrition leads to a decline in IL-10-producing F4/80^+^CD11b^+^ macrophages in the small intestine. (**a**) LPMCs were isolated from the SI of TPN- or sham-treated mice. Isolated LPMCs (5 × 10^6^ cells/ml) were then cultured for 13 hrs with or without LPS stimulation (100 ng ml^−1^). Data are given as mean ± SEM (N = 5). *P < 0.05; **P < 0.01; NS, not significant by Student’s *t*-test. (**b**) *IL-10*^Venus^ reporter mice received TPN or sham-treatment. Cell subpopulations of IL-10-expressing cells (Venus^+^ cells) within the CD45^+^7-AAD^−^ population were analyzed by flow cytometry. Frequencies of IL-10-producing MHC-II^+^F4/80^+^CD11b^+^ macrophages (Mϕs) and CD3^+^CD4^+^ T cells are shown. Data are representative of 3 independent experiments. (**c**) Frequencies of total IL-10-producing leukocytes (CD45^+^7-AAD^−^), F4/80^+^CD11b^+^ Mϕs and CD3^+^CD4^+^ T cells are shown. Data are given as mean ± SEM (N = 6). **P < 0.01; NS, not significant by Student’s *t*-test.

Since we had previously shown that IL-10 expression is significantly decreased in the SI mucosa of TPN recipient mice^16^,^17^, we first assessed IL-10 secretion by SI LP mononuclear cells (LPMCs) *ex vivo*. LPMCs were isolated from the SI of TPN- and sham-treated mice, and IL-10 production by LPMCs stimulated with LPS was measured by ELISA. IL-10 production by SI LPMCs was significantly blunted in TPN mice compared to sham-treated mice, whereas TNF-α production by SI LPMCs was not affected in TPN mice (Fig. 1a). In order to determine the source of IL-10 in SI LPMCs *in vivo*, we utilized IL-10-reporter mice (*IL-10*^Venus^ mice^19^,^20^). *IL-10*^Venus^ mice received either TPN or sham treatment and the IL-10-producing cell (Venus^+^ cells) populations were analyzed by flow cytometry. As shown in Fig. 1b, the major IL-10-producing cell populations in the SI LP were F4/80^+^CD11b^+^ Mϕs and CD3^+^CD4^+^ T cells (Fig. 1b). Consistent with the *ex vivo* IL-10 production results, TPN treatment significantly reduced the number of IL-10-producting cells (Venus^+^ cells) in total CD45^+^ leukocytes *in vivo* (Fig. 1c). Notably, IL-10Venus^+^ F4/80^+^CD11b^+^ Mϕs were significantly decreased in TPN recipient mice, while there was no change in the number of IL-10Venus^+^ CD3^+^CD4^+^ T cells (Fig. 1c). These data indicate that the decline of IL-10 production in the SI LP of TPN-treated mice is due to a lower number of IL-10-producing F4/80^+^CD11b^+^ Mϕs.

### Both replenishment of resident macrophages and their IL-10 production were impaired in TPN mice

**Figure 2:**
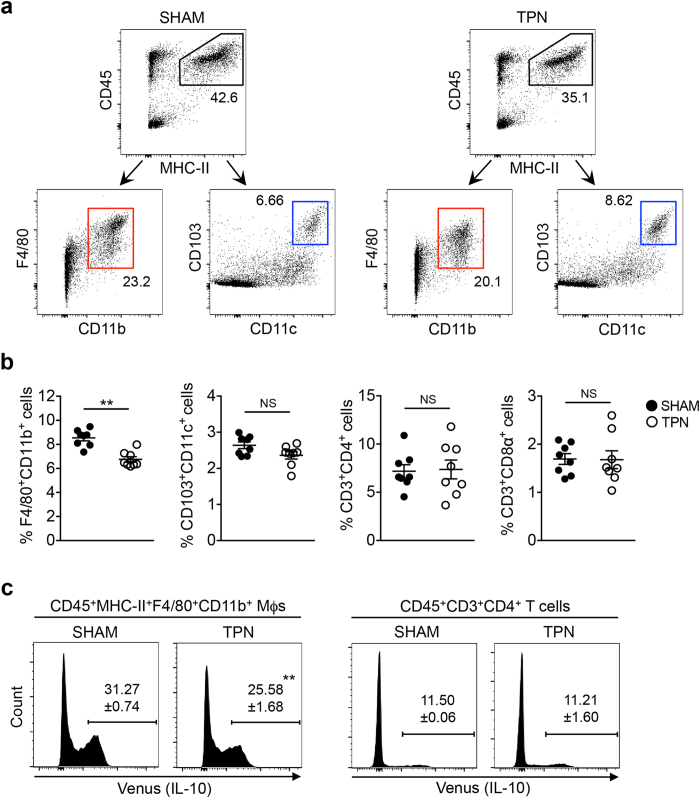
Dietary nutrients regulate replenishment of resident macrophages and their IL-10 production in the small intestine. (**a**) LPMCs were isolated from the SI of TPN- or sham-treated mice and then analyzed by flow cytometry. CD45^+^MHC-II^+^ antigen presenting cells were further gated either as F4/80^+^CD11b^+^ Mϕs or CD103^+^CD11c^+^ DCs. The numbers adjacent to the outlined area indicate the frequencies of cells above. Data are representative of 3 independent experiments. (**b**) Frequencies of F4/80^+^CD11b^+^ Mϕs, CD103^+^CD11c^+^ DCs, CD3^+^CD4^+^ T cells, and CD3^+^CD8α^+^ T cells are shown. Data are given as mean ± SEM (N = 8). **P < 0.01; NS, not significant by Student’s *t*-test. (**c**) Representative histograms of (% of Venus^+^ cells) in CD45^+^MHC-II^+^F4/80^+^CD11b^+^ Mϕs and CD45^+^CD3^+^CD4^+^ T cells. The numbers adjacent to the lines indicate the percentages of IL-10-Venus^+^ cells above. Data are given as mean ± SEM (N = 3). **P < 0.01 by Student’s *t*-test. At least 3 independent experiments produced similar results.

Next, we asked whether other antigen presenting cells (APCs), such as dendritic cells (DCs), are influenced by the lack of enteral nutrition associated with TPN. Total LPMCs were isolated from the SI of TPN- and sham-treated mice, and the proportion of Mϕ and DC subpopulations^6^,^21^ within the total number of APCs (CD45^+^MHC-II^+^ cells) was further analyzed. Interestingly, the number of F4/80^+^CD11b^+^ Mϕs, but not CD103^+^CD11c^+^ DCs, selectively declined after TPN treatment (Fig. 2a,b). Neither CD3^+^CD4^+^ nor CD3^+^CD8^+^ T cell populations were affected by the absence of enteral nutrients (Fig. 2b). These results suggest that enteral nutrient deprivation might selectively impact the number of intestinal Mϕs, but not intestinal DCs or T cells, in the SI. Furthermore, IL-10 expression gated on F4/80^+^CD11b^+^ Mϕs, but not CD3^+^CD4^+^ T cells, was selectively diminished after TPN treatment (Fig. 2c), indicating that not only the number, but also IL-10 production by F4/80^+^CD11b^+^ Mϕs is impaired by deprivation of enteral nutrients.

### CCL2/CCR2-dependent recruitment of macrophage precursors is not impaired in TPN mice

**Figure 3:**
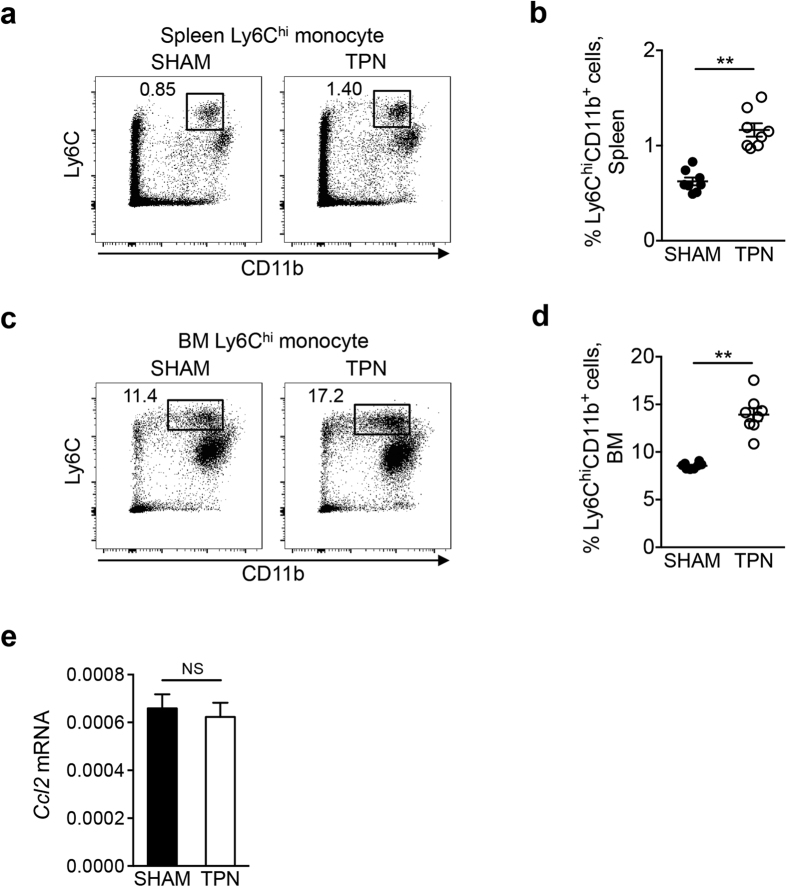
CCL2/CCR2-dependent recruitment of monocytes is not impaired by depletion of dietary antigens. Frequencies of Ly6C^hi^CD11b^+^ monocytes in the spleen (**a**,**b**) and bone-marrow (BM) (**c**,**d**) of TPN- and sham-treated mice. Representative FACS plots (**a**,**c**) and pooled results (**b,d**) are shown. Data shown are mean ± SEM (N = 8). **P < 0.01 by Student’s *t*-test. (**e**) Expression of *Ccl2* mRNA in the SI mucosa of TPN and Sham. Data shown are mean ± SEM (N = 4); NS, not significant by Student’s *t*-test.

Previous studies have demonstrated that colonic Mϕs require constant replenishment by circulating Ly6C^hi^ monocytes in order for the population to be properly maintained^5^,^10^. Ly6C^hi^ monocytes express a chemokine receptor CCR2. CCR2 is required for the migration of Ly6C^hi^ monocytes from the bone-marrow (BM) to peripheral blood^8^,^9^,^22^. CCR2-dependent recruitment of Ly6C^hi^ monocytes to the intestine is critical for the maintenance of resident macrophages in both the small intestinal and colonic LP, as the number of resident Mϕs in both the SI and the colon was significantly reduced in mice deficient in *Ccr2* or its ligand *Ccl2*^23^,^24^,^25^. To investigate the effect of enteral nutrient deprivation on the homeostasis of precursors of intestinal Mϕs in the BM and blood, we examined the number of Ly6C^hi^ monocytes in the BM and the spleen after TPN. The number of Ly6C^hi^ monocytes in TPN-treated mice was not decreased compared to sham mice (Fig. 3a–d), suggesting that enteral nutrients are not required for the homeostasis of intestinal Mϕ precursors. We next assessed the expression of *Ccl2* in the SI mucosa after TPN treatment. As is shown in Fig. 3e, the expression levels of *Ccl2* mRNA did not decrease in TPN mice. These data suggest that enteral nutrient deprivation does not affect the CCR2-dependent replenishment of intestinal Mϕs in the SI LP.

### TPN-associated dysbiosis is not involved in the decrease of small intestinal macrophages

**Figure 4:**
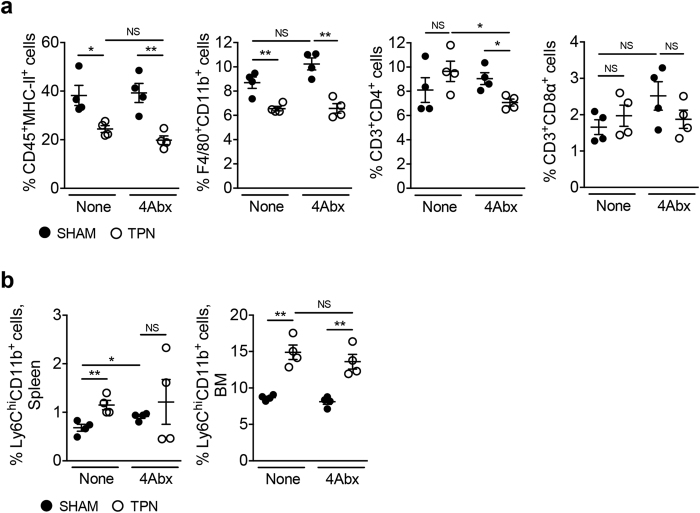
Gut microbiota is not involved in the impairment of SI macrophage homeostasis caused by enteral nutrient deprivation. (**a**) Frequencies of CD45^+^MHC-II^+^ APCs, F4/80^+^CD11b^+^ Mϕs, CD3^+^CD4^+^ T cells and CD3^+^CD8α^+^ T cells in the SI LP of TPN and sham mice with or without antibiotic treatment (4Abx). Data shown are mean ± SEM (N = 4). *P < 0.05; **P < 0.01; NS, not significant by Student’s *t*-test. (**b**) Frequencies of Ly6C^hi^CD11b^+^ monocytes in the spleen and BM isolated from TPN and sham mice with or without antibiotic treatment (4Abx). Data shown are mean ± SEM (N = 4). *P < 0.05; **P < 0.01; NS, not significant by Student’s *t*-test.

It has been reported that the presence of the gut microbiota plays a crucial role in the replenishment of Mϕs in the colon^9^,^10^. In this context, enteral nutrient deprivation is known to influence the structure of the gut microbiota^26^. Indeed, we have previously reported that the deprivation of enteral nutrition results in alterations in the composition of the resident microbiota communities in the SI, including an abnormal accumulation of Proteobacteria^27^. Therefore, it is possible that TPN-associated dysbiosis may influence resident Mϕ homeostasis in the SI LP. To test this hypothesis, we depleted the commensal microbiota with a well-established cocktail of 4 antibiotics (4Abx) with different spectra of activity^28^,^29^ (Supplementary Fig. 1). We examined the number of intestinal Mϕs in microbiota-depleted TPN and sham recipient mice by flow cytometry. Even after depleting the SI commensal bacteria, enteral nutrient deprivation decreased the number of intestinal Mϕ (sham-4Abx vs TPN-4Abx in Fig. 4a), suggesting that TPN-associated dysbiosis does not affect the number of intestinal Mϕs in the SI. It is noteworthy that the depletion of commensal microbiota did not affect the replenishment of SI Mϕ (sham-control vs sham-4Abx in Fig. 4a) in contrast to that of colonic Mϕs^9^,^10^. Furthermore, the numbers of Ly6C^hi^CD11b^+^ monocytes, which are the precursors of Mϕs, did not vary in the systemic organs of microbiota-depleted mice (Fig. 4b). Thus, the replenishment of SI Mϕs is likely not regulated by the microbiota.

### Dietary amino acids regulate the homeostasis of resident macrophages in the SI

**Figure 5:**
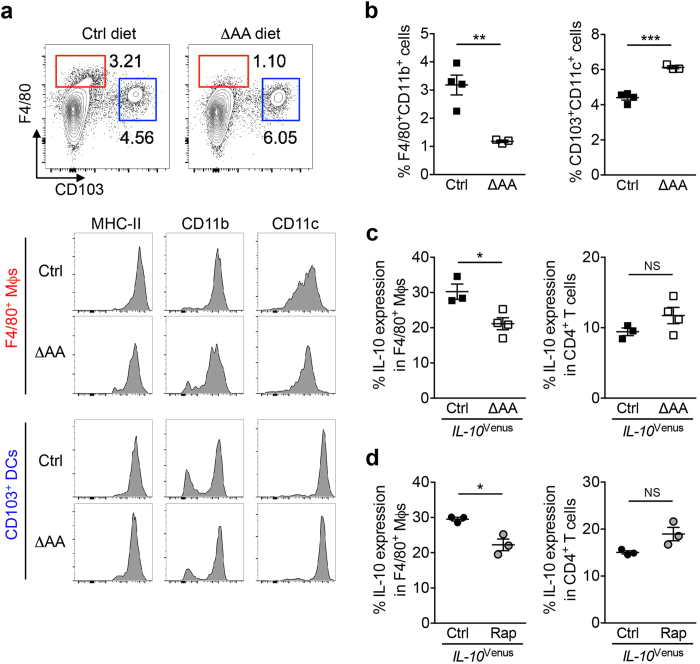
Dietary amino acids regulate SI macrophage homeostasis. (**a**) SI LPMCs were isolated from control (Ctrl) diet- or protein-free (ΔAA) diet-fed mice and analyzed by flow cytometry. F4/80^+^ and CD103^+^ cells within CD45^+^7-AAD^−^ cells are shown. Expression of MHC-II, CD11b, and CD11c on either F4/80^+^ cells (Mϕs) or CD103^+^ cells (DCs) is shown in histogram. (**b**) Frequencies of F4/80^+^CD11b^+^ Mϕs and CD103^+^CD11c^+^ DCs are shown. Data are given as mean ± SEM (Ctrl; N = 4, ΔAA; N = 3) **P < 0.01; ***P < 0.001 by Student’s *t*-test. (**c,d**) IL-10 expression (% of Venus^+^ cells) in CD45^+^MHC-II^+^F4/80^+^CD11b^+^ Mϕs and CD45^+^CD3^+^CD4^+^ T cells in the SI LPMCs isolated from Ctrl diet- and ΔAA diet-fed mice (**c**) or isolated from rapamycin-treated (Rap) or untreated control (Ctrl) mice (**d**). Data are given as mean ± SEM. *P < 0.05; NS, not significant by Student’s *t*-test.

Since TPN-induced dysbiosis is not involved in dysregulation of Mϕ homeostasis in the SI, we next focused on how direct stimulation by luminal dietary factors affects SI Mϕs. Our previous report demonstrated that enteral supplementation with a single amino acid (glutamine) can reverse the pathogenic phenotypes of TPN recipients by restoring epithelial barrier function^30^. Based on this finding, we focused on the role of dietary amino acids in the regulation of immune cells, including Mϕs, in the SI. Wild-type (WT) mice were fed with either a control (Ctrl) diet or a protein-free (ΔAA) diet for 14 days. SI LPMCs were isolated from Ctrl and ΔAA diet-fed mice, and LP Mϕs and DCs were analyzed by flow cytometry. As we expected, dietary deprivation of amino acids significantly reduced the number of F4/80^+^CD11b^+^ Mϕs (including both CD11c^hi^ and CD11c^lo^ Mϕs) in the SI LP (Fig. 5a,b). In contrast, the number of CD103^+^CD11c^+^ DCs (including both CD11b^+^ and CD11b^−^ DCs) was not reduced in ΔAA diet-fed mice (Fig. 5a,b). These results indicate that dietary amino acids regulate the homeostasis of SI Mϕ, but not DCs. Consistent with the results obtained from TPN-treated mice, dietary amino acid deprivation affected neither the number of intestinal Mϕ precursors in the BM and spleen nor *Ccl2* expression in the SI mucosa (Supplementary Fig. 2). Next, we used *IL-10*^Venus^ mice to examine the expression of IL-10 in F4/80^+^CD11b^+^ Mϕs and CD3^+^CD4^+^ T cells in the SI LPMCs isolated from Ctrl diet- and ΔAA diet-fed mice. As is shown in Fig. 5c, deprivation of dietary amino acids selectively decreased IL-10 expression in F4/80^+^CD11b^+^ Mϕs and also resulted in a decline of the total number of IL-10^+^F4/80^+^CD11b^+^ Mϕs in the SI (Fig. 5c and Supplementary Fig. 3a). In contrast, deprivation of dietary amino acids did not affect IL-10 production by T cells (Fig. 5c and Supplementary Fig. 3a). It is well known that amino acid signaling is upstream of mammalian target of rapamycin (mTOR), a conserved Ser/Thr kinase that is part of the mTOR complex 1^31^. In order to confirm whether the phenotype observed in ΔAA diet-fed mice is due to diminished stimulation from amino acids, we next attempted to block the amino-acid-mediated mTOR activation pathway by treatment with an mTOR inhibitor rapamycin^32^. As expected, IL-10 expression in F4/80^+^CD11b^+^ Mϕs and the total number of IL-10^+^F4/80^+^CD11b^+^ Mϕs in the SI were selectively reduced in rapamycin-treated mice (Fig. 5d and Supplementary Fig. 3b). These results suggest that stimulation from dietary amino acids plays an important role in the regulation of resident Mϕ homeostasis in the SI.

## Discussion

Our results show a strong association between enteral nutrient, particularly amino acid, deprivation and the impaired replenishment of intestinal Mϕs and their IL-10 production in the SI. These results provide important and novel insights into the mechanism by which enteral nutrient deprivation leads to inflammatory mucosal changes in the SI^16^,^17^. IL-10 produced by intestinal Mϕs plays a key role in the dampening of microbial-induced pro-inflammatory immune responses in the intestine at steady-state^2^,^6^,^7^. Intestinal resident Mϕs spontaneously produce robust amounts of IL-10 and are known to be tolerant to microbial stimulation^4^. For instance, intestinal Mϕs fail to produce pro-inflammatory cytokines, such as TNF-α, IL-6, IL-12, and IL-23 in response to TLR ligands, such as LPS^33^. In contrast, intestinal Mϕs isolated from *Il10*^−/−^ mice are capable of producing the pro-inflammatory cytokines upon stimulation with TLR ligands^34^, indicating IL-10 plays a key role in this tolerogenic phenotype. Enteral nutrient deprivation due to TPN results in a loss of epithelial barrier function, thereby causing the translocation of microbial components, such as LPS, released from the enteric microbiota^35^,^36^,^37^,^38^. A decreased number of IL-10-producing Mϕs in the SI LP may cause uncontrolled immune responses against these disseminated microbial components, thereby eliciting intestinal inflammation in TPN-treated mice as well as patients^13^. Although we have demonstrated that enteral nutrient deprivation compromises intestinal Mϕ homeostasis in the SI, the precise mechanisms by which this phenotype is triggered remain unaddressed. Similar to other tissue resident Mϕs, intestinal Mϕs arise from embryonic precursors and self-renew locally during the fetal period^10^. On the other hand, in the adult mouse, the embryonic precursor-derived Mϕs are gradually replaced with Ly6C^hi^ monocyte-derived Mϕs in the intestine^5^,^7^,^10^,^21^. Eventually, the vast majority of intestinal resident Mϕs become monocyte-derived^10^,^21^. Since the monocyte-derived intestinal Mϕ cannot proliferate locally, a constant replenishment by recruited Ly6C^hi^ monocytes is required for the maintenance of intestinal Mϕ homeostasis^9^,^10^,^39^. The CCL2-CCR2 axis is a key pathway involved in the migration of Ly6C^hi^ monocytes from the BM to peripheral blood^8^,^9^,^22^. Although the mechanisms that control the relocation of peripheral blood Ly6C^hi^ monocytes into the intestine are not fully elucidated^40^, the CCL2-CCR2 pathway is critical for the replenishment of monocyte-derived Mϕs in the intestine^8^,^9^,^22^. Indeed, the lack of this pathway (i.e., in *Ccr2*^−/−^ or CCR2-DTR mice) results in a marked decrease of intestinal Mϕs in both the SI and colon^23^,^24^,^25^. Our results demonstrate that enteral nutrient deprivation selectively affects the monocyte-derived F4/80^+^CD11b^+^ intestinal Mϕs population regulated by CCL2-CCR2, but does not impact the homeostasis of CD103^+^CD11c^+^ DCs, which arise from non-monocytic precursor cells^1^. This fact implies that enteral nutrient deprivation influences the CCL2-dependent monocytes recruitment pathway. However, CCL2 expression in the SI mucosa is not affected by the deprivation of enteral nutrition. Interestingly, the numbers of Ly6C^hi^ monocytes in the BM and the spleen are even higher in TPN-treated mice compared to sham mice. These results indicate there is a possibility that unimpaired CCL2 signaling in the intestine of TPN mice sends a signal to monocytes, instructing them to relocate from the BM to peripheral blood. However, the entry of monocytes from blood to the intestinal LP is somehow impaired in TPN mice; therefore, the emerging monocytes stay and accumulate systemically. Further work is required to determine the mechanisms by which peripheral blood monocytes enter the intestinal LP, and how these mechanisms are affected by the absence of enteral nutrients. Another possible explanation why peripheral monocytes are more abundant in TPN mice is due to TPN-triggered translocation of microbial components. It has been reported that enteral nutrient deprivation caused by TPN increases the permeability of intestinal epithelium, thereby causing the translocation of microbial components^35^,^36^,^37^,^38^. Systemically disseminated microbial components of luminal origin may promote the differentiation of monocytes in the BM, as it has been reported that activation of TLR signaling skews the differentiation of hematopoietic stem cells toward the monocytic lineage^41^,^42^.

In the colonic LP, the replenishment of Mϕs is tightly regulated by the microbiota^9^,^10^. The number of colonic Mϕs in germ-free or antibiotic-treated mice is significantly reduced compared to mice with a conventional microbiota^7^,^10^. Moreover, IL-10 production by intestinal Mϕs is also regulated by the microbiota. The microbiota can directly stimulate intestinal Mϕs, likely through TLRs, to induce IL-10^7^,^43^,^44^. In addition to direct stimulation, metabolites generated by the gut microbiota, such as butyrate and niacin, are capable of inducing IL-10 by activating GPR109a receptor in intestinal Mϕs^45^. Initially we thought that enteral nutrient deprivation due to TPN impairs the homeostasis of IL-10-producing Mϕ in the SI through modification of the gut microbiota. Indeed, TPN treatment significantly alters the composition of microbial communities in the SI, since dietary factors are important energy sources for the resident microbiota^26^. However, contrary to our prediction, depletion of the microbiota by treatment with broad-spectrum antibiotics did not influence the outcome of TPN treatment in the SI, indicating that the gut microbiota is not a major factor in the control of Mϕ homeostasis (replenishment and IL-10 production) in the SI. Consistently, there was no difference in the SI Mϕ populations between germ-free and SPF mice (Supplementary Fig. 4). What else then could possibly regulate the replenishment of Mϕs in the SI? Since recruited Ly6C^hi^ monocytes constantly differentiate *in situ* into intestinal Mϕs, we assessed the expression of *Csf1*, a growth factor that drives this differentiation^5^, in the SI mucosa of TPN and sham-treated mice. Neither CSF-1 nor CSF-2, a growth factor that induces the differentiation of DCs^1^, mRNA levels were altered after enteral nutrient deprivation (Supplementary Fig. 5), suggesting that the decline in monocyte-derived intestinal Mϕs in TPN mice is not due to a defect of differentiation. Another possibility is that dietary factors directly regulate the homeostasis of Mϕs in the SI. Although many of known immune-regulatory diet-derived luminal metabolites, such as short-chain fatty acids (SCFAs), bile acids, and vitamins, require processing by the gut microbiota (e.g., fermentation of dietary fiber for SCFAs, conversion from primary to secondary bile acids, microbial enzymes needed to generate vitamins), certain nutrients derived from the diet can directly, without processing by the microbiota, modulate immune cell functions in the intestine^46^,^47^,^48^. For instance, a recent study has revealed that the development of immune cells in the SI is regulated by dietary antigens^49^. Using the dietary antigen-free mouse model, in which the animals were fed a liquid elemental diet, the authors were able to show that memory-phenotype CD4^+^ T cells were significantly decreased in the SI, although CD4^+^ T cell counts in the colon were normal. These data indicate that local activation of CD4^+^ T cells in the SI is driven mainly by dietary antigens, whereas in the colon it is induced by the microbiota^49^. Notably, in their report, protein antigens in the diet are important for inducing the development of a subset of peripherally-raised T_reg_ (pT_reg_) cells in the SI. Furthermore, mice fed an amino acid-diet (contains no intact protein) developed a similar phenotype as those on a liquid elemental diet^49^. Dietary antigen-induced regulatory immunity in the SI is known to prevent mucosal immune responses against food antigens, thereby limiting the development of food allergies^49^. Perhaps not surprisingly, the impact of dietary antigens on host immune development/regulation has attracted considerable attention in recent years. However, the approaches that utilize low-antigenicity diets, such as the elemental diet or the amino acid-diet, have limitations. For example, although the use of elemental diet can in part address the impact of dietary nutrients on host immune regulation, this diet contains simple sugars, amino acids and other essential nutrients, and the effects of these nutrient factors cannot be ignored in the context of this model. It has been reported that dietary amino acids modulate intestinal immunity^50^,^51^ despite amino acids being less immunogenic than intact proteins. Likewise, most dietary macromolecules, including proteins, are degraded by the time they reach the SI. Therefore, micromolecules derived from food, such as amino acids, represent the majority of diet-derived nutritional factors in the SI. As such, food-derived micromolecules, such as amino acids, may also effect on host immune regulation like food-derived macromolecules, such as intact proteins. Our approach using the TPN model allowed us to completely remove dietary factors from the GI tract without causing systemic malnutrition, thus making it possible to test the impact of whole dietary factors, including micromolecules. Using this model, we have found that IL-10-producing F4/80^+^ Mϕs in the SI are regulated by dietary antigens. This phenotype is not observed in mice fed with the elemental diet^49^, indicating that micromolecules contained in the elemental diet contribute to the regulation of intestinal Mϕs in the SI. We decided to focus on the role of dietary amino acids in the regulation of intestinal Mϕs in the SI, since accumulating evidence suggests that dietary amino acids are capable of modulating intestinal immune cell functions^52^,^53^. As expected, mice fed the ΔAA diet displayed similar phenotypes as those observed in TPN-treated mice in terms of replenishment of SI LP Mϕs and Mϕ-derived IL-10 production. These results indicate that dietary amino acids might be involved in the regulation of Mϕ homeostasis in the SI. However, a general lack of dietary amino acids might cause systemic malnutrition and affect several organ systems. Systemic malnutrition might have unintended effects on the intestinal immune system, including Mϕ homeostasis. In order to overcome this potential obstacle, we attempted to inhibit the mTOR-mediated amino acid sensing. To this end, we treated mice with an mTOR inhibitor rapamycin. As expected, inhibition of the mTOR pathway affected homeostasis of intestinal Mϕs, as can be seen in the case of TPN treatment and ΔAA diet. This suggests that stimulation from dietary amino acids might play a key role in the regulation of Mϕ homeostasis in the SI. However, it remains unclear whether dietary amino acids act directly on intestinal Mϕs or rather affect other cell types, such as epithelial cells, and the activation of these cells, in turn, regulates Mϕ function. In this context, we have demonstrated that either the absence of amino acids from the cell culture medium or inhibition of the mTOR pathway by rapamycin selectively reduced IL-10 production by BM-derived Mϕs *in vitro* (Supplementary Fig. 6a,b). Although the mode of action of dietary amino acids *in vivo* remains uncertain, these results indicate there is a possibility that dietary amino acids act directly on intestinal Mϕs. A previous study demonstrated that CD11c^+^ cell-specific depletion of regulatory-associated protein mTOR (raptor) impairs IL-10 production by intestinal CD11c^+^ Mϕ/DC populations^54^, supporting the notion that intestinal Mϕ/DC populations directly sense amino acids *in vivo*. Another unaddressed question is which amino acids are responsible for the regulation of the Mϕ homeostasis. In order to address this question, we examined whether single amino acid supplementation could restore IL-10 production by Mϕs (Supplementary Fig. 6c). However, none of the amino acids, when administered individually, sufficiently restored IL-10 production in Mϕ (Supplementary Fig. 6c). Further studies are needed to clarify the mechanism by which dietary amino acids regulate Mϕ function and to elucidate which amino acids control this function *in vitro* and *in vivo*.

Although we have demonstrated that the lack of dietary antigens results in impaired intestinal Mϕ homeostasis, dietary antigens may also regulate other immune cell functions. As noted above, a recent report showed that administration of a low antigenicity diet (e.g., elemental diet or amino acid-diet) leads to a decline in Foxp3^+^RORγt^−^ pT_reg_ cells^49^. The same report also notes that dietary antigens regulate homeostasis of CD103^+^CD11b^+^ DCs^49^. We did not make such an observation in either TPN-treated or ΔAA diet-fed mice during the course of our study. There are multiple explanations that could address these contradictory results. In our study, we only focused on the production of IL-10 by CD4^+^ T cells and did not analyze the various subsets of T_reg_ cells. While dietary antigens are required for the induction of Foxp3^+^RORγt^−^ pT_reg_ cells, the generation of Foxp3^+^RORγt^+^ pT_reg_ cells is regulated by the gut microbiota, even in the SI^49^. Another possible explanation why TPN affects Mϕs and not T_reg_ cells can be found in the difference of their respective *in vivo* life span. The turnover of intestinal resident Mϕs is more rapid than that of T_reg_ cells. Intestinal Mϕs are constantly replenished by circulating blood monocytes^10^. Therefore, deprivation of dietary antigens for 5 days due to TPN or 2 weeks due to ΔAA diet is sufficient to affect the number of Mϕs. In contrast, T_reg_ cells are relatively stable compared to Mϕs and it takes longer than 2 weeks for an animal deprived of dietary antigens to experience a decline in the number of T_reg_ cells in the SI^49^. Likewise, the absence of dietary antigens may influence the homeostasis of LP DCs. We did not observe a reduction in the number of CD103^+^CD11c^+^ DCs in TPN-treated mice; however, we only assessed the frequency of total CD103^+^CD11c^+^ cells and did not analyze the CD11b^+^ and CD11b^−^ subsets as was done by others. Since it has been demonstrated that administration of a low antigenicity diet results in a decrease of CD103^+^CD11b^+^ DCs and an increase in the number of CD103^+^ CD11b^−^ DCs^49^, it is conceivable that the impact of dietary antigen deprivation on total CD103^+^ DCs (including both CD11b^+^ and CD11b^−^) becomes negligible, as was observed by us. It is noteworthy that dietary antigen deprivation brought about by TPN and low antigenicity or ΔAA diets may influence the number as well as function of other immune and non-immune cells (e.g. epithelial cells) in the SI in addition to the above mentioned cell types. Further studies are required to better understand the role of dietary antigens in the development and regulation of the mucosal immune system.

Although the gut microbiota is not involved in the decline of IL-10-producing Mϕ in TPN-treated mice, our previous reports demonstrated that the microbiota plays a central role in adverse phenotype expression seen in TPN-treated mice (i.e., increased epithelial permeability, pro-inflammatory cytokine up-regulation in SI mucosa), as the lack of TLR4 and MyD88, an adaptor molecule downstream of most TLRs^55^, reverses the pathogenic phenotypes in TPN recipient mice^27^,^56^. Likewise, previous work has shown that TPN administration leads to a perturbation of intestinal bacteria, shifting from a Firmicutes-dominant population to a Proteobacteria-dominant population in the SI^27^,^57^. Since Proteobacteria are known to be a major source of LPS (TLR4 ligand) and Firmicutes lack this ligand, TPN-induced dysbiosis may be associated with the adverse phenotypes of TPN-treated mice. These facts indicate that enteral nutrient deprivation leads to at least two distinct events: (i) compromised replenishment of IL-10-producing Mϕ in the SI, and (ii) the microbial composition shifts toward more immune-stimulatory (TLR4 ligand rich). Although these two events may occur in parallel, they will eventually act in concert to augment the pathophysiology of TPN-induced adverse effects, as it has been reported that IL-10 production by intestinal Mϕ is critical to limiting pro-inflammatory responses induced by the microbial ligands, such as LPS^4^.

In conclusion, enteral nutrient deprivation due to TPN leads to a profound decline in the number of IL-10-producing Mϕ in the SI. Unlike colonic Mϕ, Mϕ in the SI do not require stimulation from the resident microbes in order to be constantly replenished. Rather, direct stimulation from dietary amino acids contribute to the regulation of homeostasis of IL-10-producing Mϕ in the SI. Supplementation of certain key nutrients through the enteral route may prevent disruption of SI homeostasis in patients receiving TPN. Moreover, since intestinal Mϕs and Mϕ-derived IL-10 production play crucial roles in limiting mucosal immune responses against food antigens as well as commensal microbial antigens, a better understanding of the impact dietary amino acids have on the regulation of intestinal Mϕs will result in novel treatments for food allergies as well as inflammatory bowel disease.

## Methods

### Mice

WT C57BL/6 mice were obtained from the Jackson Laboratory (Bar Harbor, ME) and IL-10 reporter (*IL-10*^Venus^) mice on a C57BL/6 background were generated as previously described^19^. 8 to 12 weeks old male mice were used in all experiments. All mice were housed in the same room under specific pathogen-free conditions in the Animal Facility at the University of Michigan where they had been allowed to acclimate for 1 week prior to surgery. In some experiments mice were switched from conventional animal chow to either a control diet (amino acid control diet; TD.130595, Harlan) or a protein-free diet (TD.93328, Harlan) (Supplementary Table 1), and fed with these custom diets for 14 days. All animal studies were performed in accordance with protocols approved by the University Committee on Use and Care of Animals (UCUCA) at the University of Michigan.

### Depletion of microbiota

Animals were treated with antibiotics as previously described^28^,^29^. Briefly, we used an antibiotic cocktail (4Abx) consisting of ampicillin (1 g l^−1^), neomycin (1 g l^−1^), metronidazole (1 g l^−1^) and vancomycin (500 mg l^−1^). The antibiotics were administered in drinking water when the mice reached the age of 9 weeks, starting 4 days before surgery for a total of 7 days. After 7 days the animals received sterilized drinking water until sacrificed (5 days later).

### A mouse model of total parenteral nutrition

WT or *IL-10*^Venus^ or microbiota-depleted mice underwent jugular vein cannulation as previously described^15^,^58^. Mice were allowed to recover for 1 day after cannulation with full access to chow and water while receiving 5 ml/d of 0.9% saline through the catheter. On day 2, mice in the study group had their chow removed and started receiving a balanced parenteral nutrition solution as previously described^27^,^58^ (Supplementary Table 2). All mice were housed in individually vented cage racks. All mice were sacrificed on day 7 by CO_2_ asphyxiation.

### Rapamycin treatment

Rapamycin (LC Laboratories, Woburn, MA) was reconstituted in Dimethyl Sulphoxide Hybri-Max (Sigma-Aldrich, St. Louis, MO), and then diluted in 5% Tween-80 (Sigma-Aldrich) and 5% Polyethylene glycol (PEG) 400 (Hampton Research, Aliso Viejo, CA). Mice received rapamycin (4 mg kg^−1^ ip) every other day for 2 weeks^32^.

### Isolation of lamina propria mononuclear cells

LPMCs were isolated from the SI specimens using a modification of a previously described protocol^21^,^59^. Briefly, the dissected mucosal tissue was incubated in calcium and magnesium-free Hank’s balanced salt solution (HBSS) (Life technologies, Carlsbad, CA) containing 2.5% heat-inactivated fetal bovine serum (FBS) (Life technologies) and 1 mM dithiothreitol (Sigma-Aldrich) to remove mucus. The mucosa was then incubated with agitation in HBSS containing 2 mM and 1 mM EDTA (Quality Biological, Gaithersburg, MD) for 15 min and 45 min, respectively, at 37 °C. The tissues were then collected and incubated with agitation in HBSS containing 400 U ml^−1^ of type 3 collagenase and 0.01 mg ml^−1^ of DNase I (Worthington Biochemical, Lakewood, NJ) for 90 min at 37 °C. The insoluble fraction was pelleted, re-suspended in a 40% Percoll solution (GE Healthcare Life Sciences, Pittsburgh, PA), layered on top of a 75% Percoll solution and centrifuged at 2,000 r.p.m. for 20 min at room temperature. Viable LPMCs were recovered from the discontinuous gradient interface.

### Isolation of spleen and bone marrow cells

The spleens were collected, mashed and digested with 200 U ml^−1^ of type 3 collagenase and 0.01 mg ml^−1^ DNase I (Worthington Biochemical) for 30 min at 37 °C. The BM was collected from mouse femurs and tibias. After centrifugation, both spleen and BM cell pellets were lysed with 1× RBC lysis buffer (eBioscience, San Diego, CA) to remove residual red blood cells and the remaining cells were analyzed by flow cytometry.

### Preparation of BM derived macrophages

BM cells were isolated from mouse femurs and tibias. After isolation, BM mononuclear cells were cultured for 7 days in Iscove’s Modified Dulbecco’s Medium (IMDM) containing 10% FBS, 1% penicillin/streptomycin, 2-mercaptoethanol (25 μM), sodium pyruvate (1 mM), MEM non essential amino acids (NEAA) (all from Life technologies) and 30% L-cell conditioned medium. After differentiation, cells were collected and washed two times with PBS^59^.

### Measurement of cytokine production

LPMCs were re-suspended in complete RPMI medium containing 10% FBS, 1% penicillin/streptomycin, 2-mercaptoethanol (50 μM), L-glutamine (2 mM), sodium pyruvate (1 mM), HEPES (1 mM) and MEM non-essential amino acids (NEAA) (all from Life technologies) at 5 × 10^6^ cells per ml. LP cells were then incubated for 13 hours at 37 °C with LPS (100 ng ml^−1^) or PBS (Life technologies). BM derived Mϕs (BMDMs) were incubated at 1 × 10^6^ cells per ml in amino acid containing RPMI (Ctrl RPMI) or amino acid deficient RPMI (ΔAA RPMI) (Supplementary Table 3) or were incubated at 2 × 10^6^ cells per ml in pretreated with or without 25 ng ml^−1^ rapamycin for 1 hour in complete RPMI^54^, followed by stimulation with 100 ng ml^−1^ LPS for 24 hours. Culture supernatants were harvested and cytokine levels were measured by ELISA.

### Quantitative real-time PCR

Mucosal scrapings were placed in TRIzol reagent (Life Technologies), homogenized and used for RNA extraction according to the manufacturer’s instructions. Purified RNA was reverse-transcribed into cDNA as previously described^60^. The cDNA was then used for quantitative real-time PCR (RT qPCR) in combination with SYBR Green Supermix (BioRad, Hercules, CA) using a Rotor-Gene 6000 (Corbett Life Science, Sydney, Australia). The fold changes of target genes were calculated using comparative quantification with β-actin as reference gene. For bacterial quantification, small intestinal tissue and stool were harvested from the terminal ileum, homogenized mechanically and used for DNA extraction using E.Z.N.A. Stool DNA kit (OMEGA bio-tek, Norcross, GA). RT-qPCR was performed using SYBR Green PCR master mix (Alkali Scientific, Pompano Beach, FL) and the StepOne Real-time PCR system (Applied Biosystems). The fold changes of 16S rDNA gene were calculated and normalized to genome β-actin^61^,^62^. Primer sequences are provided in Supplementary Table 4.

### Flow cytometry

Flow cytometry was performed using the LSR Fortessa or FACS Canto II (BD Bioscience) and analyzed using FlowJo software (Tree Star, Ashland, OR). Dead cells were excluded by 7-AAD staining. Non-specific antibody binding was blocked with anti CD16/32 antibody. Fluorescence-conjugated mAb against CD11b (M1/70), CD11c (N418), F4/80 (BM8), Ly6C (HK1.4), MHC class II (I-A/I-E) (M5/114.15.2), CD103 (2E7), CD45 (30-F11), CD3 (17A2), CD4 (GK1.5), CD8α (53.67) were from eBioscience (Supplementary Table 5). Isotype-matched antibodies (eBioscience) were used for control staining. All antibodies were used at 1:200 dilution except CD11b (used in 1:100 dilution) and Ly6C, MHC class II, CD45 (used in 1:1000 dilution). The concentration of cell suspension was adjusted to 1 × 10^6^ cells per 100 μl.

### Statistical analysis

Statistical analyses were performed using GraphPad Prism software version 6.0 (GraphPad Software). Student’s *t*-test (parametric) was used to assess significance between two populations. Data are presented as mean ± SEM, unless otherwise specified. Differences at *P* < 0.05 were considered significant.

## Additional Information

**How to cite this article**: Ochi, T. *et al*. Diet-dependent, microbiota-independent regulation of IL-10-producing lamina propria macrophages in the small intestine. *Sci. Rep.* **6**, 27634; doi: 10.1038/srep27634 (2016).

## Supplementary Material

Supplementary Information
