# Supplementary material for: Diet-dependent, microbiota-independent regulation of IL-10-producing lamina propria macrophages in the small intestine
Source: Sci Rep. 2016 Jun 15;6:27634. doi: 10.1038/srep27634 (PMC4908404; doi:10.1038/srep27634)
Supplement: Supplementary Information [file srep27634-s1.pdf]

Supplementary Information

Diet-dependent, microbiota-independent regulation of IL-10-producing lamina propria macrophages in the small intestine

Takanori Ochi, Yongjia Feng, Sho Kitamoto, Hiroko Nagao-Kitamoto, Peter Kuffa, Koji Atarashi, Kenya Honda, Daniel H. Teitelbaum, Nobuhiko Kamada

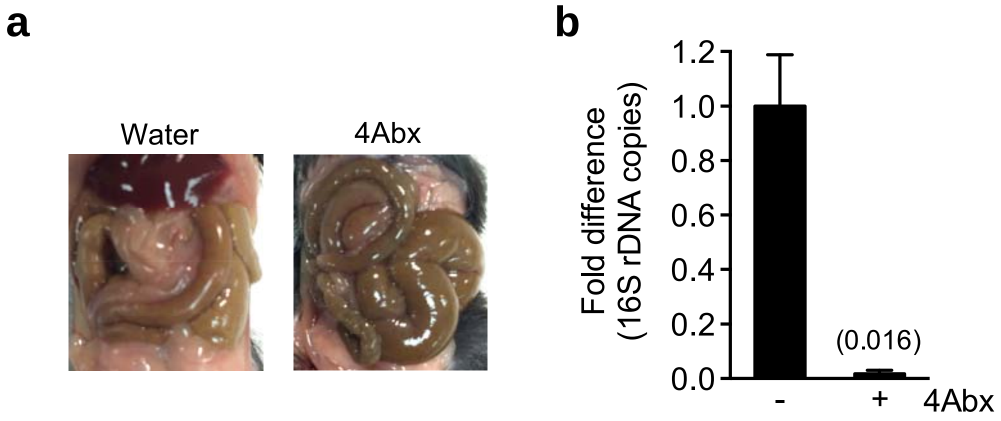

Supplementary Figure 1. Antibiotics treatment efficiently decreases the number of the small intestinal microbes.
SPF C57BL/6 mice received an antibiotic cocktail (4Abx) in the drinking water for 7 days. Mice were then switched to sterile water for 5 days before analysis of ileal bacteria. Representative images of cecum from 4Abx-treated and control mice are shown in (a). (b) Luminal contents were isolated from terminal ileum and bacterial genomic DNA was isolated. qPCR was performed and the fold changes of 16S rDNA (normalized to host genomic *Actb)* are shown.

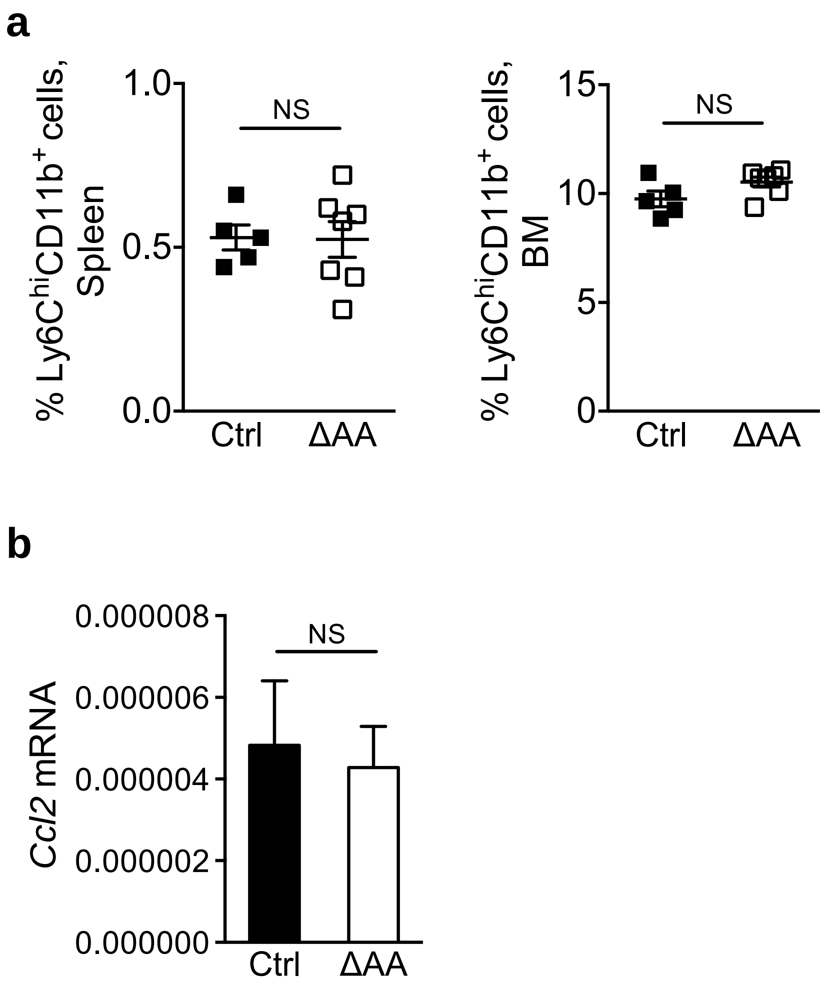

Supplementary Figure 2. A lack of dietary amino acids does not affect the number of macrophage precursors and mucosal *Ccl2* expression.
(a) Frequencies of $Ly6C^{hi}CD11b^{+}$ monocytes in the spleen and the bone-marrow (BM) isolated from control (Ctrl) diet- or protein-free (ΔAA) diet-fed mice. Data are given as mean ± s.e.m. (Ctrl; n=5, ΔAA; n=7). NS, not significant by Student's *t*-test.
(b) Expression of *Ccl2* mRNA in the small intestinal mucosa of Ctrl diet- or Δ AA diet-fed mice. Data are given as mean ± s.e.m. (n=4). NS, not significant by Student's *t*-test.

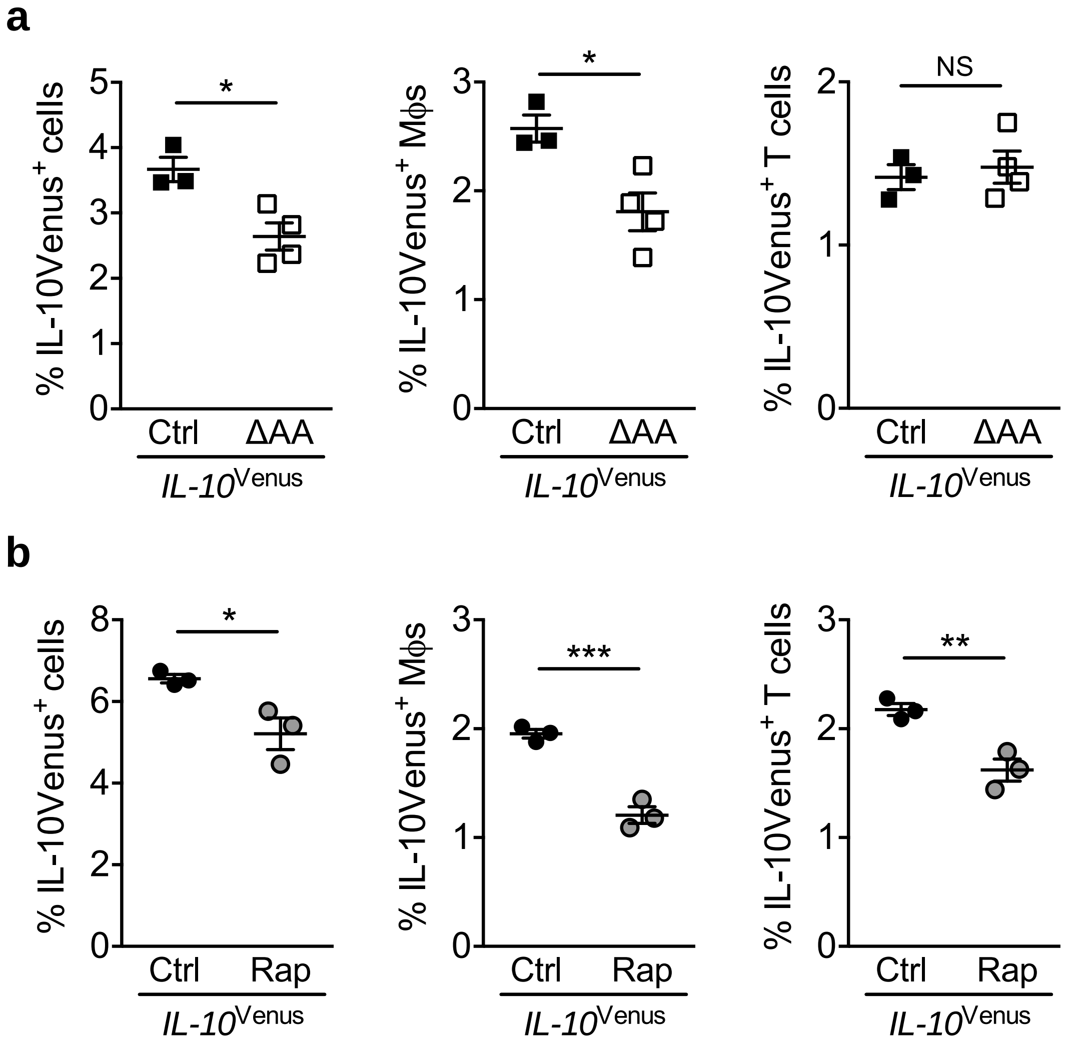

Supplementary Figure 3. Dietary amino acid deprivation or rapamycin treatment decreases the number of IL-10-producing macrophages.
(a, b) Frequencies of total IL-10-producing leukocytes ($CD45^+7\text{-}AAD^-$), $F4/80^+CD11b^+$ Mϕs and $CD3^+CD4^+$ T cells in the SI LPMCs isolated from control (Ctrl) diet- or protein-free (ΔAA) diet-fed mice (a), or isolated from rapamycin-treated (Rap) or untreated control (Ctrl) mice (b) are shown. Data are given as mean±s.e.m. *, P<0.05; **, P<0.01; ***, P<0.001; NS, not significant by Student's t-test.

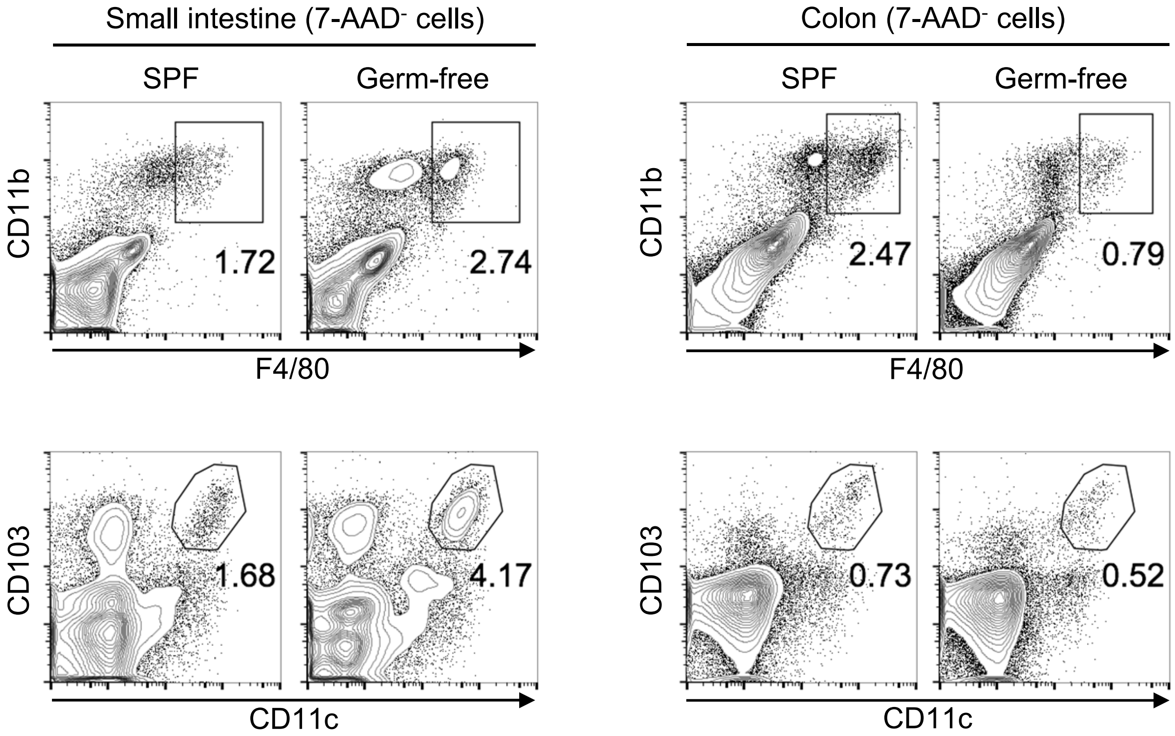

Supplementary Figure 4. Gut microbiota is not required for the replenishment of F4/80$^+$CD11b$^+$ macrophages in the small intestine.

Lamina propria mononuclear cells were isolated from the small intestine and colon of SPF and germ-free mice. Frequencies of F4/80$^+$CD11b$^+$ cells (monocyte-derived macrophages) and CD103$^+$CD11c$^+$ cells (dendritic cells) within total lamina propria cells (7-AAD$^-$) are shown. Data are representative of at least 2 independent experiments.

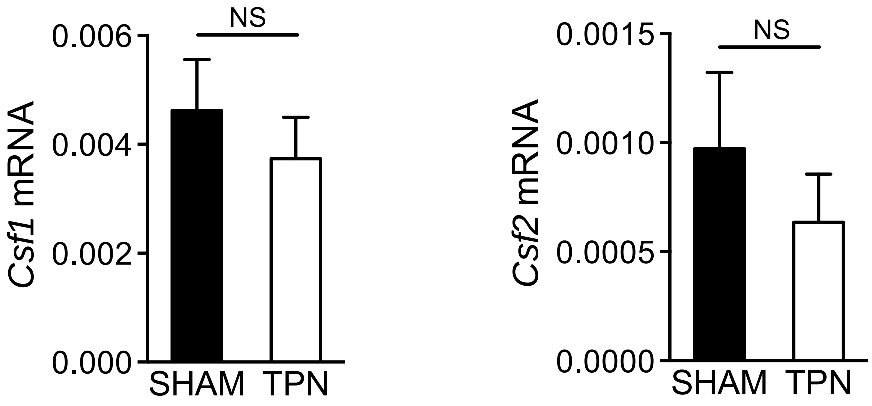

Supplementary Figure 5. Expression of *Csf1* and *Csf2* mRNA in the small intestinal mucosa of TPN-treated mice.

Mucosal samples were isolated from the small intestine of TPN- or sham-treated mice. Expression of *Csf1* and *Csf2* mRNA was analyzed by qPCR. Expression of target genes was normalized to *Actb*. Data are given as mean±s.e.m. (n=6). NS, not significant by Student's *t*-test.

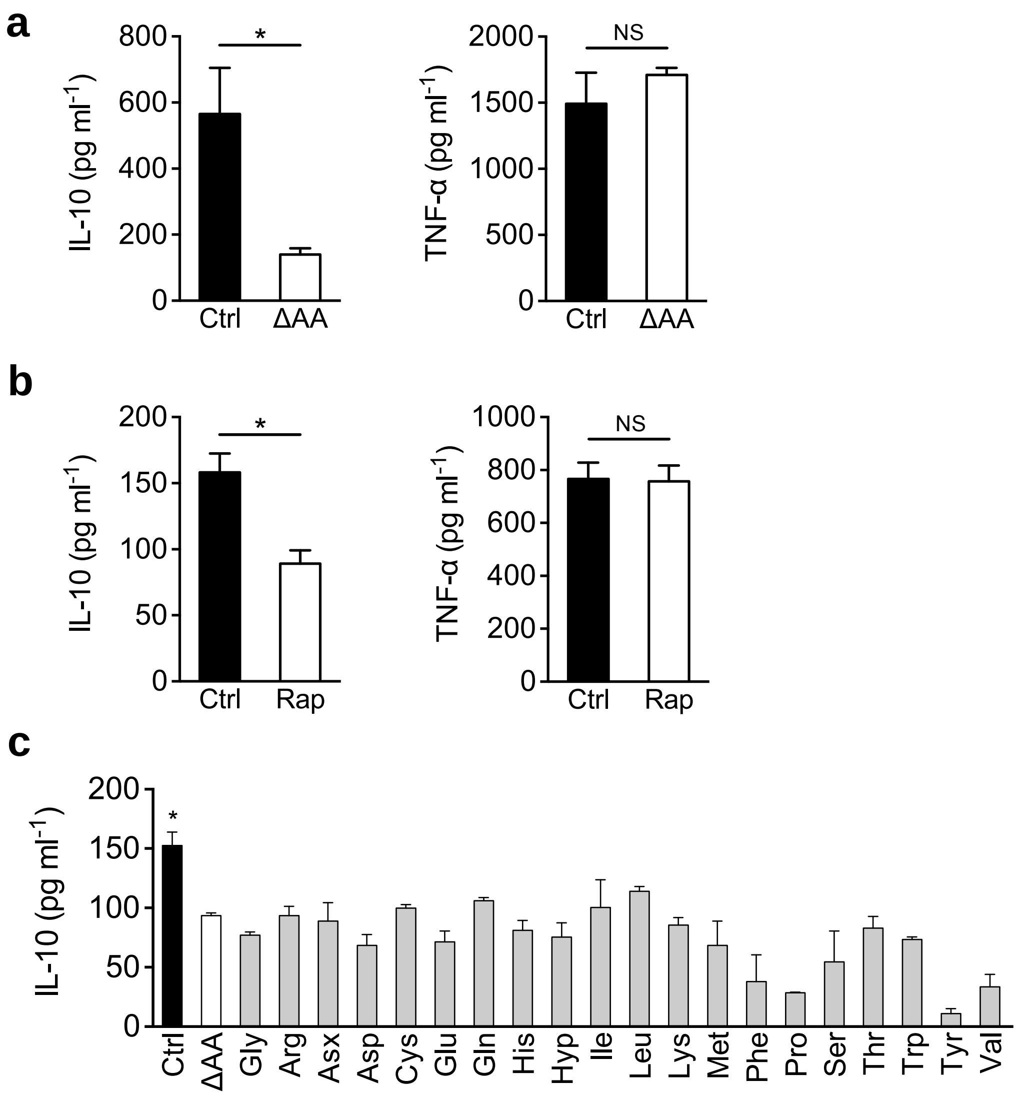

Supplementary Figure 6. Amino acid deprivation leads to a decrease of IL-10 production in bone-marrow derived macrophages.

(a) Bone-marrow (BM) derived macrophages (BMDMs) (1×10^6 cells ml^{-1}) were differentiated in complete RPMI. Cells were washed and medium was replaced with either an amino acid (AA) containing RPMI (Ctrl) or AA deficient (ΔAA) medium (formulation of each medium is listed in Supplementary Table 3), and then stimulated with LPS (100ng ml^{-1}) for 24 hrs. Cytokine levels were measured by ELISA. Data are given as mean ± s.e.m. (n=3). *, P<0.05; NS, not significant by Student's *t*-test. (b) BMDMs (in complete RPMI) were pretreated with or without 25 ng ml^{-1} rapamycin (Rap) for 1 hr followed by stimulation with LPS (100ng ml^{-1}) for 24 hrs. Cytokine levels were measured by ELISA. Data are given as mean ± s.e.m. (n=3). *, P<0.05; NS, not significant by Student's *t*-test. (c) BMDMs were cultured in Ctrl or ΔAA medium supplemented with indicated individual amino acids (final conc. 1mM). LPS (100ng ml^{-1}) for 24 hrs. Secreted IL-10 was measured by ELISA. Data are given as mean ± s.e.m. (n=3). *, P<0.05 by Dunnett's test (compared to ΔAA).

Supplementary Table 1. Composition of amino acid control diet and protein-free diet.

amino acid control diet

Formula	Amount (g/Kg)
Sucrose	452.2
Corn Starch	200.0
Corn Oil	54.6
Cellulose	66.462
Mineral Mix, Ca-P Deficient	13.37
Calcium Phosphate, dibasic	23.72
Calcium Carbonate	0.038
Vitamine Mix, Teklad	10.0
Ethoxyquin, antioxidant	0.01
L-Alanine	3.5
L-Arginine HCl	12.1
L-Asparagine	6.0
L-Aspartic Acid	3.5
L-Cystine	3.5
L-Glutamic Acid	40.0
Glycine	23.3
L-Histidine HCl, monohydrate	4.5
L-Isoleucine	8.2
L-Leucine	11.1
L-Lysine HCl	18.0
L-Methionine	8.2
L-Phenylalanine	7.5
L-Proline	3.5
L-Serine	3.5
L-Threonine	8.2
L-Tryptophan	1.8
L-Tyrosine	5.0
L-Valine	8.2

protein-free diet

Formula	Amount (g/Kg)
Sucrose	631.8
Corn Starch	200.0
Corn Oil	54.6
Cellulose	66.462
Mineral Mix, Ca-P Deficient	13.37
Calcium Phosphate, dibasic	23.72
Calcium Carbonate	0.038
Vitamine Mix, Teklad	10.0
Ethoxyquin, antioxidant	0.01

Supplementary Table 2. Composition of the TPN solution.

Nutrient	Intake over 24 h
Dextrose, g[1]	0.26
Amino Acids, g[2]	0.04
Fat, g[3]	0.02
Sodium, mmol	0.47
Potassium, mmol	0.97
Chloride, mmol	0.34
Acetate, mmol	1.06
Phosphate, mmol	0.10
Magnesium, mmol	0.03
Calcium, mmol	0.06
Sulfate, mmol	0.02
Gluconate, mmol	0.10
Heparin, U[1]	36.0
MTE-5 Concentrate, mL[4]	0.007
MVI-12, mL[5]	0.070
Energy, kJ	35.87

[1]Hospira, Deerfield, Illinois.

[2]FreAmine III (B.Braun) was used as the source of the following amino acids (mg/100ml): Essential: isoleucine 6.60; Leucine 10.00; Lysine (acetate) 10.50; methionine 1.72; phenylalanine 2.98; thronine 4.00; tryptophan 2.00; valine 5.00. Nonessential: alanine 9.93; arginine 10.18; L-aspartic acid 7.00; L-glutamic acid 7.38; histidine 3.00; praline 7.22; serine 5.30; N-acetyl-L-tyrosine 2.70; glycine 5.00.

[3]Intravenous fat solution (Intralipid, Baxter Healthcare) is composed of (g/L): safflower oil 50; soybean oil 50; egg phosphatides 12; glycerin in water 25. The major fatty acid components (%) are: linoleic (18:2, n-6) 65.8; oleic (18:1, n-9) 17.7; palmitic (16:0) 8.8; stearic (18:0) 3.4; linolenic (18:3, n-3) 4.2.

[4]American Regent Laboratories, Shirley, NY, MTE-5 (multi-trace 5) contains (amount given per 24 hours): zinc sulfate heptahydrate 0.155 mg; cupric sulfate pentahydrate 0.028 mg; manganese sulfate monohydrate 0.011 mg; chronic chloride hexahydrate 0.359 mg; selenious acid 0.686 mg.

[5]For MVI-12 (Hospira; amount given per 24 hours): Vitamin A (retinol) 0.0014 mg; Vitamin D (ergocalciferol) 0.0070 µg; Vitamin E (di-alpha-tocopheryl acetate) 0.0140 mg; Vitamin C (ascordbic acid) 0.280 mg; Niacinamide 0.056 mg; Vitamin B_2 (riboflavin 5-phosphate sodium) 0.0050 mg; Vitamin B_1 (thiamine) 0.0084 mg; Vitamin B_6 (pyridoxine HCl) 0.0084 mg; Dexpanthenol (di-pantothenyl alchol) 0.021 mg; Biotin 0.084 µg; Folic Acid 0.840 µg; Vitamin B_{12} (cyanocobalamin) 0.0070 µg.

Supplementary Table 3. Composition of amino acid (AA) Ctrl RPMI and AA deficient (ΔAA) RPMI.

Type	Nutrients	Amount (mM)	Ctrl RPMI	ΔAA RPMI
Amino Acids	Glycine	0.13333334	O	-
	L-Arginine	1.1494253	O	-
	L-Asparagine	0.37878788	O	-
	L-Aspartic acid	0.15037593	O	-
	L-Cystine 2HCl	0.20766774	O	-
	L-Glutamic acid	0.13605443	O	-
	L-Glutamine	2.0547945	O	-
	L-Histidine	0.09677419	O	-
	L-Hydroxyproline	0.15267175	O	-
	L-Isoleucine	0.3816794	O	-
	L-Leucine	0.3816794	O	-
	L-Lysine hydrochloride	0.21857923	O	-
	L-Methionine	0.10067114	O	-
	L-Phenylalanine	0.09090909	O	-
	L-Proline	0.17391305	O	-
	L-Serine	0.2857143	O	-
	L-Threonine	0.16806723	O	-
	L-Tryptophan	0.024509804	O	-
	L-Tyrosine disodium salt dihydrate	0.11111111	O	-
	L-Valine	0.17094018	O	-
Vitamins	Biotin	8.20E-04	O	
	Choline chloride	0.021428572	O	
	D-Calcium pantothenate	5.24E-04	O	
	Folic Acid	0.002267574	O	
	Niacinamide	0.008196721	O	
	Para-Aminobenzoic Acid	0.00729927	O	
	Pyridoxine hydrochloride	0.004854369	O	
	Riboflavin	5.32E-04	O	
	Thiamine hydrochloride	0.002967359	O	
	Vitamin B12	3.69E-06	O	
	i-Inositol	0.19444445	O	
Inorganic Salts	Calcium nitrate (Ca(NO3)2 4H2O)	0.42372882	O	
	Magnesium Sulfate (MgSO4-7H2O)	0.407	O	
	Potassium Chloride (KCl)	5.3333335	O	
	Sodium Bicarbonate (NaHCO3)	23.809525	O	
	Sodium Chloride (NaCl)	103.44827	O	
	Sodium Phosphate dibasic (Na2HPO4-7H2O)	5.633803	O	
Other Components	D-Glucose (Dextrose)	11.111111	O	
	Glutathione (reduced)	0.003257329	O	
	Phenol Red	0.013283741	O	

Note: For vitamins, inorganic salts and other components, a single O is centered across the Ctrl RPMI and ΔAA RPMI columns.

* Bovine serum albumin (BSA) (20 mg ml^{-1}) was added instead of FBS.

Supplementary Table 4. Primer sequences used in this study.

Gene	Forward (5' -> 3')	Reverse (5' -> 3')
Ccl2	GTTGGCTCAGCCAGATGCA	AGCCTACTCATTGGGATCATCTTG
Csf1	GGGGGCCTCCTGTTCTAC	CCCACAGAAGAATCCAATGTC
Csf2	ATGCCTGTCACGTTGAATGAAG	GCGGGTCTGCACACATGTTA
Actb	AAGTGTGACGTTGACATCCG	GATCCACATCTGCTGGAAGG
16S rDNA	AGAGTTTGATCCTGGCTCAG	TGCTGCCTCCCGTAGGAGT
Actb (genome)	ATGACCCAGATCATGTTTGA	TACGACCAGAGGCATACAG

Supplementary Table 5. List of antibodies used for flow cytometry.

Anti-Mouse CD11b FITC (eBioscience 11-0112)
Anti-Mouse CD11b PE (eBioscience 12-0112)
Anti-Mouse CD103 (Integrin alpha E) PE (eBioscience 12-1031)
Anti-Mouse CD11c PE-Cyanine7 (eBioscience 25-0114)
Anti-Mouse CD4 PE-Cyanine7 (eBioscience 25-0041)
Anti-Mouse F4/80 Antigen APC (eBioscience 17-4801)
Anti-Mouse CD3 APC (eBioscience 17-0032)
Anti-Mouse CD45 APC-eFluor 780 (eBioscience 47-0451-80)
Anti-Mouse MHC Class II (I-A/I-E) eFluor 450 (eBioscience 48-5321-80)
Anti-Mouse CD8a eFluor 450 (eBioscience 48-0081-80)
Anti-Mouse Ly-6C eFluor 450 (eBioscience 48-5932)
7-AAD Viability Staining Solution (eBioscience 00-6993-50)
